# Identification of a bridge-specific intramolecular exciton dissociation pathway in donor–π–acceptor alternating conjugated polymers

**DOI:** 10.1186/s11671-021-03507-0

**Published:** 2021-03-20

**Authors:** Zhaoyong Jiao, Tingting Jiang, Zhongpo Zhou, Chaochao Qin, Jinyou Long, Yufang Liu, Yuhai Jiang

**Affiliations:** 1 Henan Key Laboratory of Infrared Materials and Spectrum Measures and Applications, School of Physics, Henan Normal University, Xinxiang, 453007 People’s Republic of China; 2 State Key Laboratory of Magnetic Resonance and Atomic and Molecular Physics, Innovation Academy for Precision Measurement Science and Technology, Chinese Academy of Sciences, Wuhan, 430071 People’s Republic of China; 3 Shanghai Advanced Research Institute, Chinese Academy of Sciences, Shanghai, 201210 People’s Republic of China

**Keywords:** Donor-π-acceptor, Conjugated polymers, Charge transfer, Exciton relaxation dynamics

## Abstract

Intramolecular exciton dissociation is critical for high efficient mobile charge carrier generations in organic solar cells. Yet despite much attention, the effects of π bridges on exciton dissociation dynamics in donor–π–acceptor (D-π-A) alternating conjugated polymers remain still unclear. Here, using a combination of femtosecond time-resolved transient absorption (TA) spectroscopy and steady-state spectroscopy, we track ultrafast intramolecular exciton relaxation dynamics in three D-π-A alternating conjugated polymers which were synthesized by Qin's group and named HSD-A, HSD-B, HSD-C. It is found that the addition of thiophene unit as π bridges will lead to the red shift of steady-state absorption spectrum. Importantly, we reveal the existence of a new intramolecular exciton dissociation pathway mediated by a bridge-specific charge transfer (CT′) state with the TA fingerprint peak at 1200 nm in π-bridged HSD-B and HSD-C. This CT′ state results in higher electron capture rates for HSD-B and HSD-C as compared to HSD-A. Depending on the proportion of CT′ state and nongeminate recombination are important step for the understanding of high power conversion efficiencies in HSD-B than in HSD-C. We propose that this bridge-specific exciton dissociation pathway plays an important role in ultrafast intramolecular exciton dissociation of organic photovoltaic material D-π-A alternating conjugated polymers.
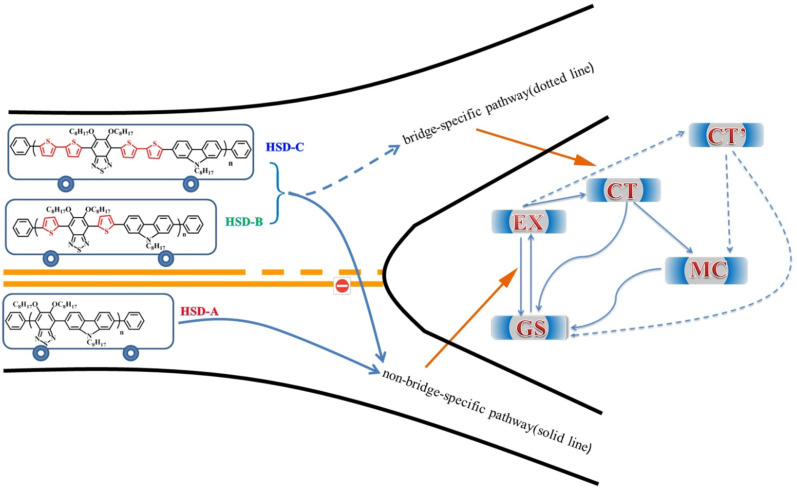

## Introduction

Organic photovoltaic (OPV) devices that use solar energy to meet the world’s growing energy demand could be regarded as one of the most momentous substitutes to clean and renewable energy sources production [1–4]. Donor–acceptor alternating conjugated polymers, in which conjugated blocks of different electron affinity are arranged alternately along the main chain of the polymers, are excellent organic electronic material. The fact that it shows a relatively high power conversion efficiencies (PCE) is partly because of the lower optical gaps that allow for more efficient collection of solar photons in the near infrared (NIR) range [5]. In consequence, OPV devices that consist of Donor–Acceptor alternating conjugated polymers could become the economically viable alternatives to Si-based solar cells [6–9].

The latest researches from various groups have shown that the efficiency of OPV can be improved by using low band gap conjugated alternating copolymers (as seen in PCDTBT, PBDTTT, and PTB families) [10–13]. An important difference between these polymers is that the exciton transition at the lowest energy level exhibits partial charge-transfer characteristics. The intramolecular charge transfer state is considered to promote the final charge separation at the heterojunction [14–19]. So it is reasonable to expect that the properties of low-bandgap donor polymers are closely related to the performance of devices. However, the connection between the performance of devices and the inherent properties of polymers is still indistinct. Specially, the ultrafast exciton splitting and carrier dynamics of low-bandgap donor polymers are not directly related to the PCE of the devices. On the one hand, a mass of parallel and sequential course on both ultrafast and slow temporal scale is only found in equipment-related conditions. On the other hand, only exciton split in the donor–acceptor bulk heterojunction (BHJ) interfacial could be considered to condition the device PCE [5]. Therefore, it is necessary to study the exciton and carrier dynamics of low-bandgap donor polymers to optimize the PCE of D-A organic photovoltaic devices.

A series of D-π-A alternating conjugated polymers have recently been synthesized by Qin's group [20–22]. For example, the HSD copolymers consist of 2,7-linked carbazole as the donor unit and 5,6-bis(octyloxy)benzo[c][1,2,5] thiadiazole as the acceptor unit, while have different π bridges. The donor block and the acceptor block are directly polymerized to prepare the photovoltaic material as HSD-A. Differently, one thiophene unit acts as the π-bridge connecting donor block and acceptor block is denoted as HSD-B, as well as the donor block and the acceptor block are connected by two thiophene units is denoted as HSD-C. They found that π bridges in the copolymers have a significant effect on the properties of HSD copolymers. Different π bridges critically affect the electron delocalization of the conjugated polymer’s main chain, the morphology of the film, and the optical, electrochemical, charge transport, and photovoltaic properties of the HSD copolymers [23]. Using HSD copolymers as electron donor and PC_71_BM as electron acceptor to prepare organic photovoltaic devices, it is found that the devices prepared with HSD polymers with different π bridges have different PCE. OPV devices with HSD-A:PC71BM as the active layer demonstrated the PCE is low; HSD-B:PC71BM exhibits a PCE of 5.4%; HSD-C:PC71BM shows a PCE of 2.15% [20, 21]. These evidences indicate that donor copolymers have an effect on PCE of polymer solar cells, but the correlation between devices performance and inherent characteristics of donor copolymers such as structure, energetic, and mobile carrier dynamics still indistinct. Major relaxation processes which follow light absorption are important for determining the performance of photovoltaic devices. Therefore, it is imperative to understand the exciton and carrier dynamics of HSD copolymers by tracking excitons.

The rapid development of ultra-short laser technology makes it possible to monitor and track the formation and breaking of chemical bonds in molecules and various dynamic processes within and between molecules with femtosecond time resolution and high spatial precision. This work elucidates the exciton dissociation and ultrafast relaxation processes of HSD copolymers using a combination of steady-state absorption and femtosecond time-resolved transient absorption spectroscopy. The characteristic spectra bands were measured and analyzed in detail, revealing an ultrafast relaxation mechanism for exciton dissociation dynamics. Our results give a better insight into the physical properties of HSD copolymers and provide an experimental basis for improving the PCE of polymer solar cells.

## Materials and experimental methods

### Materials

**Fig. 1:**
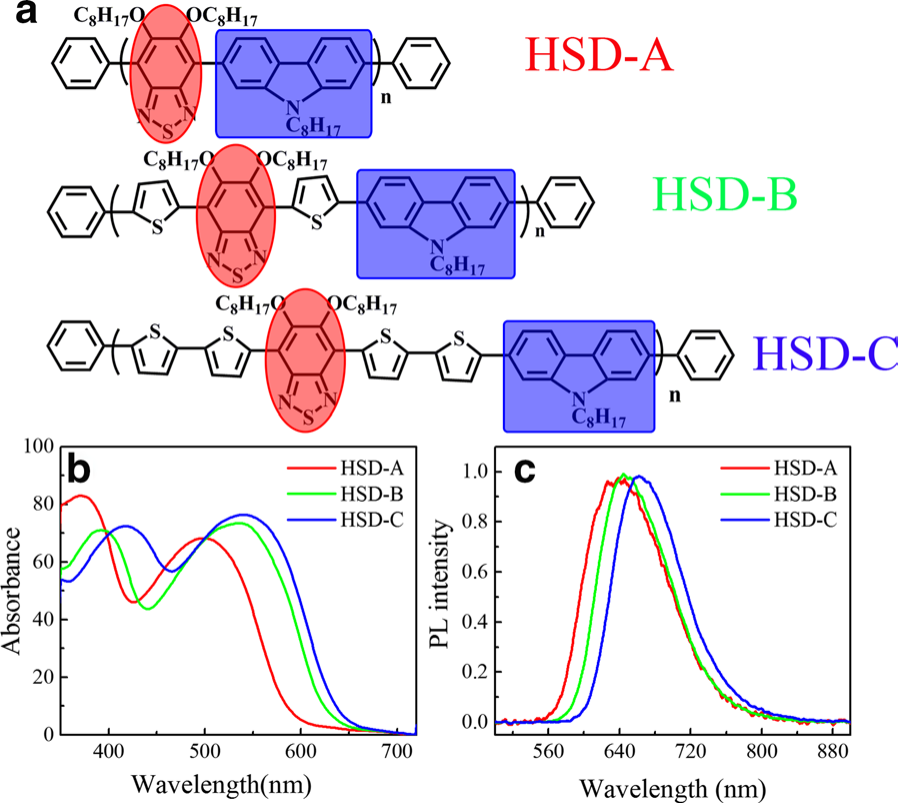
Molecular structures of three polymers (**a**) in this work. Shaded blue and shaded red denote the donor and acceptor parts, respectively. Steady-state absorption spectra (**b**) and steady-state photoluminescence spectra (**c**) of three samples, HSD-A (red), HSD-B (green), HSD-C (blue), measured in o-dichlorobenzene

HSD-A, HSD-B, HSD-C were provided from Qin's group, and the synthesis and characterization of these co-oligomers were shown in the literatures [20, 21]. The molecular structures of these co-oligomers are shown in Fig. 1a. The solution used for preparing these co-oligomers was o-dichlorobenzene, with a concentration of about 0.1 mg/ml. This concentration can not only ensure that a good time-resolved signal can be measured, but also can ensure that the chromophore is fully separated, so that the excited state will not be quenched under the excitation intensity used [24].

### Spectroscopic measurements

Steady-state absorption spectroscopy was measured by a dual-beam spectrophotometer (Cary-5000, Agilent), and the steady-state fluorescence spectroscopy was measured by a fiber optic spectrometer (USB-4000, Ocean Optics).

The femtosecond time-resolved transient absorption spectroscopy was measured by Femtosecond laser (Coherent), optical parametric amplifier (OPA, TOPAS), and transient absorption spectrometer (Helios fire). The femtosecond laser generated by femtosecond laser is divided into two paths through a beam splitter (1:1), one of which enters into TOPAS and generates pump pulses with different wavelengths; the other beam passes through a beam splitter (2:98) again, and the small part of the projected laser enters the Helios transient absorption spectrometer to generate white-light continuum (WLC) probe pulses (420–780 nm, 820–1600 nm).

## Results and discussion

**Fig. 2:**
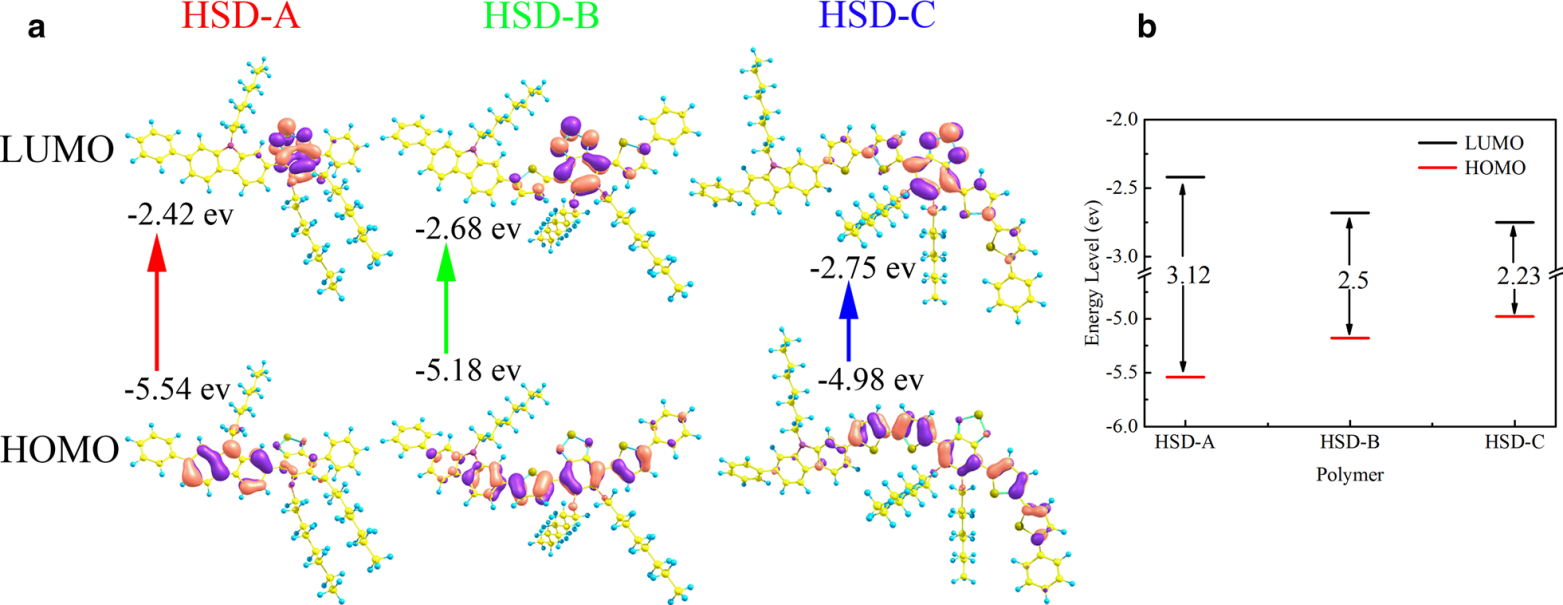
Frontier molecular orbitals of HSD copolymers (**a**) calculated using the B3LYP-D3 functional with6-311G** basis set and the HOMO and LUMO energy levels (**b**)

Figure 1a shows the structural formula of the HSD conjugated polymers used in this work, the donor moieties are marked with a blue box, and the acceptor moieties are highlighted with a red circle. The thiophene unit acts as the bridge-specific between the donor and acceptor, sequentially avoiding the steric repulsion between the individual donor and acceptor units. As previously reported, it can also achieve long-distance charge separation between the donor and acceptor, thus ensuring a long-lived charge transfer state [8]. Figure 1b exhibits the steady-state absorption spectra of the three polymers, and the absorption spectra of the three polymers with different π bridges present similar shapes, featured by two distinct absorption bands. The typical two-peak profile has also been reported in other conjugated polymers, which is unique characteristics for D-A conjugated polymers [25–30]. The absorption peaks of HSD-A are at around 370 and 490 nm, and those of HSD-B are at around 390 and 530 nm, and those of HSD-C are at around 420 and 540 nm. These two absorption peaks are attributed to π–π* transition with the lower-energy peak associated with intrachain charge transfer [31]. The positions of the steady-state absorption peaks are affected by the replacement of different π bridges units, resulting in the redshift of the absorption peaks mainly on account of the electron delocalization effect [32]. We have performed quantum chemical calculations on polymers, and the frontier molecular orbitals of HSD polymers have been calculated and provided in Fig. 2a. The highest occupied molecular orbital (HOMO) and the lowest unoccupied molecular orbital (LUMO) of the three samples are shown as Fig. 2a, and the HOMO–LUMO energy gaps (ΔE_H–L_) are plotted in Fig. 2b. It can be clearly seen from Fig. 2b that the HOMO–LUMO energy gaps (ΔE_H–L_) from HSD-A to HSD-B and HSD-C gradually decrease, which is consistent with the red shift in the spectra from HSD-A to HSD-B and HSD-C [33]. Figure 1c presents the Photoluminescence (PL) spectra of the three samples in their solution. The PL spectra of the three samples are simili-semblable, and consistent with the steady-state absorption spectra. It is noteworthy that their peaks move towards the long wave with the increase in thiophene numbers. The π bridges of HSD polymers can tune the PCE of organic solar cells fabricated by the blend of HSD polymer and PC71BM, the PCE of devices we used in this study is listed in the following order: HSD-B > HSD-C > HSD-A [20, 21].

**Fig. 3:**
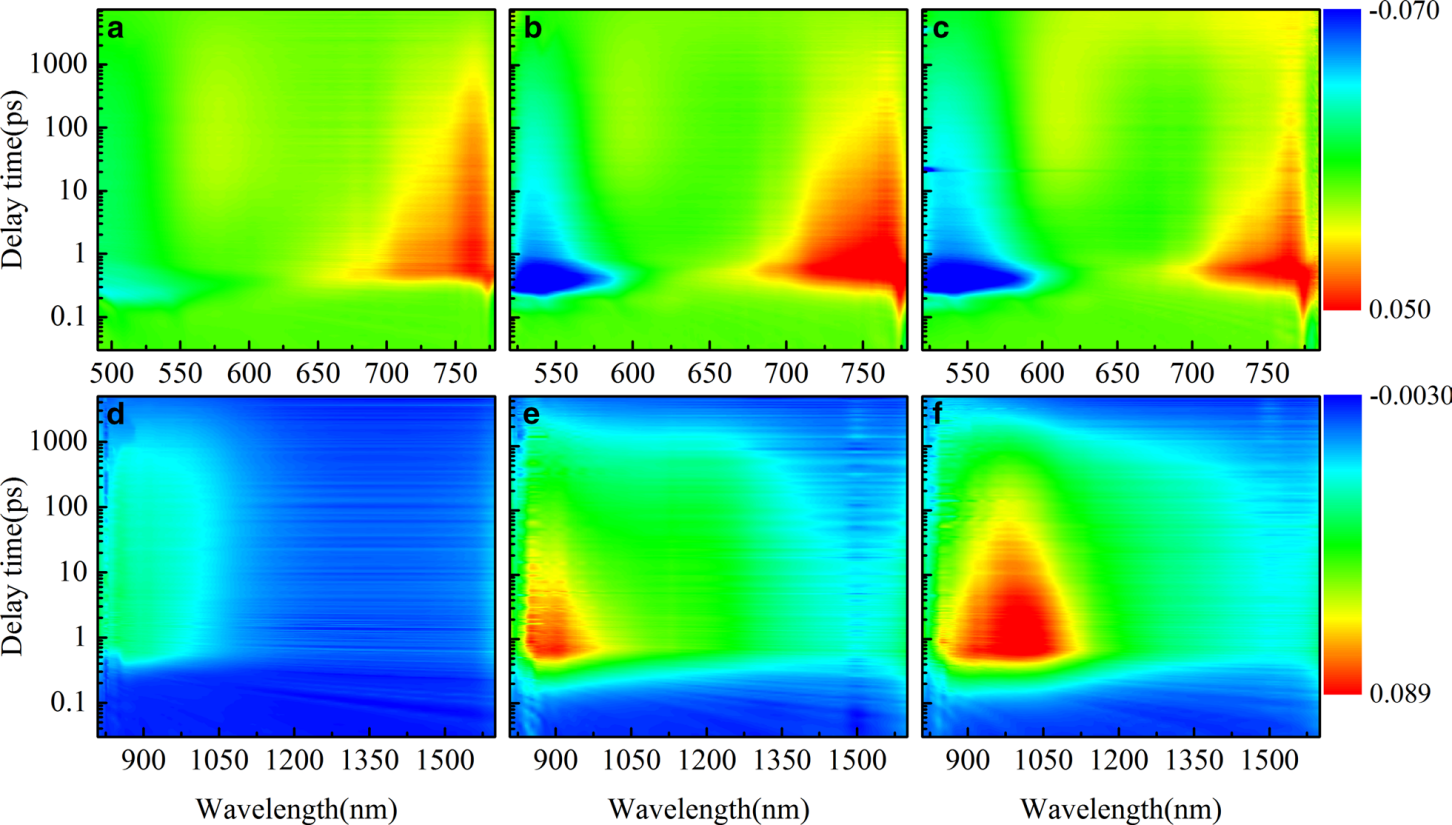
Femtosecond time-resolved transient absorption (TA) spectra at different probe wavelength of different samples. Two-dimensional maps as a function of the probe wavelength (500–1600 nm) on excitation at 470 nm wavelength of the HSD-A (**a**, **d**); excitation at 500 nm wavelength of the HSD-B (**b**, **e**); excitation at 500 nm wavelength of the HSD-C (**c**, **f**)

Steady-state spectroscopy can only give a macro-description of the overall electronic transition states. In order to investigate how π-bridges affect the PCE of devices, we further conducted transient absorption measurements of the three HSD polymers as shown in Fig. 3. The graphs of the visible (VIS) ranges of the three samples (Fig. 3a–c) are similar, showing three spectral features. The negative signal (light blue in the map) at about 500 nm is assigned to ground state bleaching (GSB) signal, because it corresponds well to the second steady absorption peak as shown in Fig. 1b. All three samples have two positive absorption signals (light red in the map) in the visible range, and the absorption peaks are at 600 nm and 750 nm, respectively, which considered as excited state absorption (ESA) [34]. In the near infrared (NIR) detection range (Fig. 3d–f), the three samples show obvious differences. HSD-A has almost no absorption signal in the near infrared range, but HSD-B and HSD-C have large-area red absorption signal within the scope of 800–1500 nm.

**Fig. 4:**
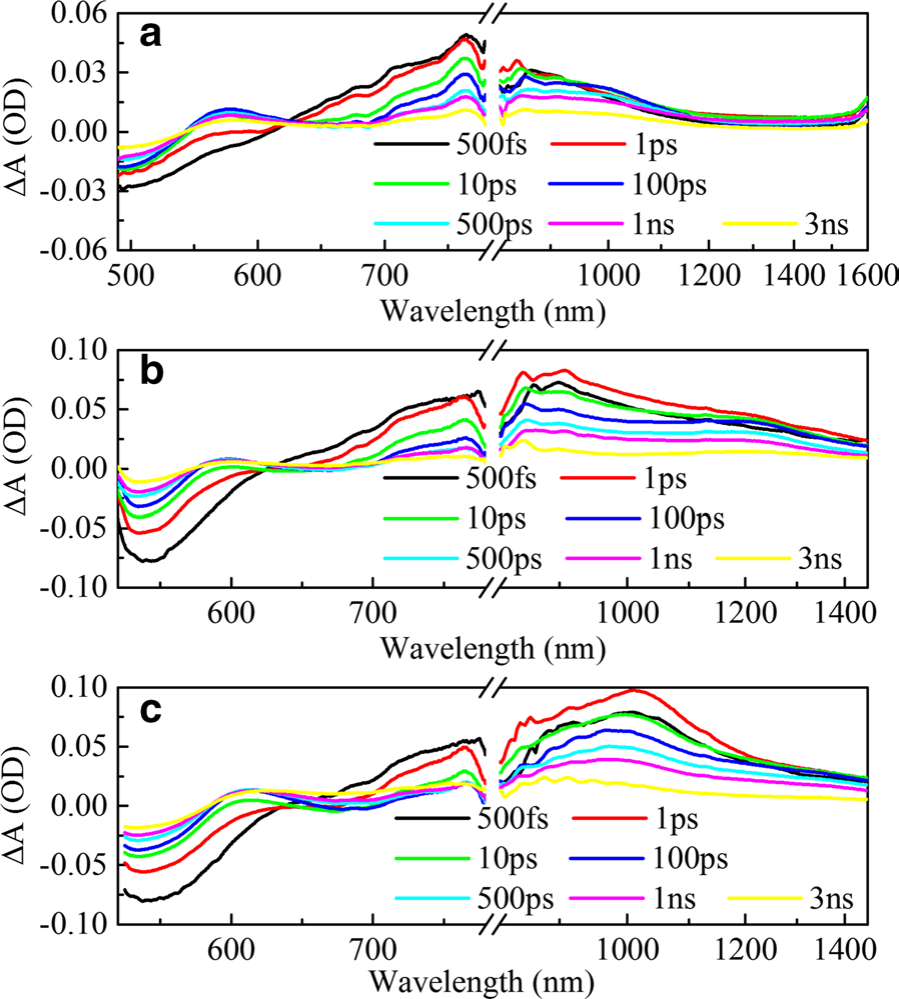
Evolution-associated difference spectra (EADS) in VIS–NIR of HSD-A (**a**), HSD-B (**b**), HSD-C (**c**)

**Fig. 5:**
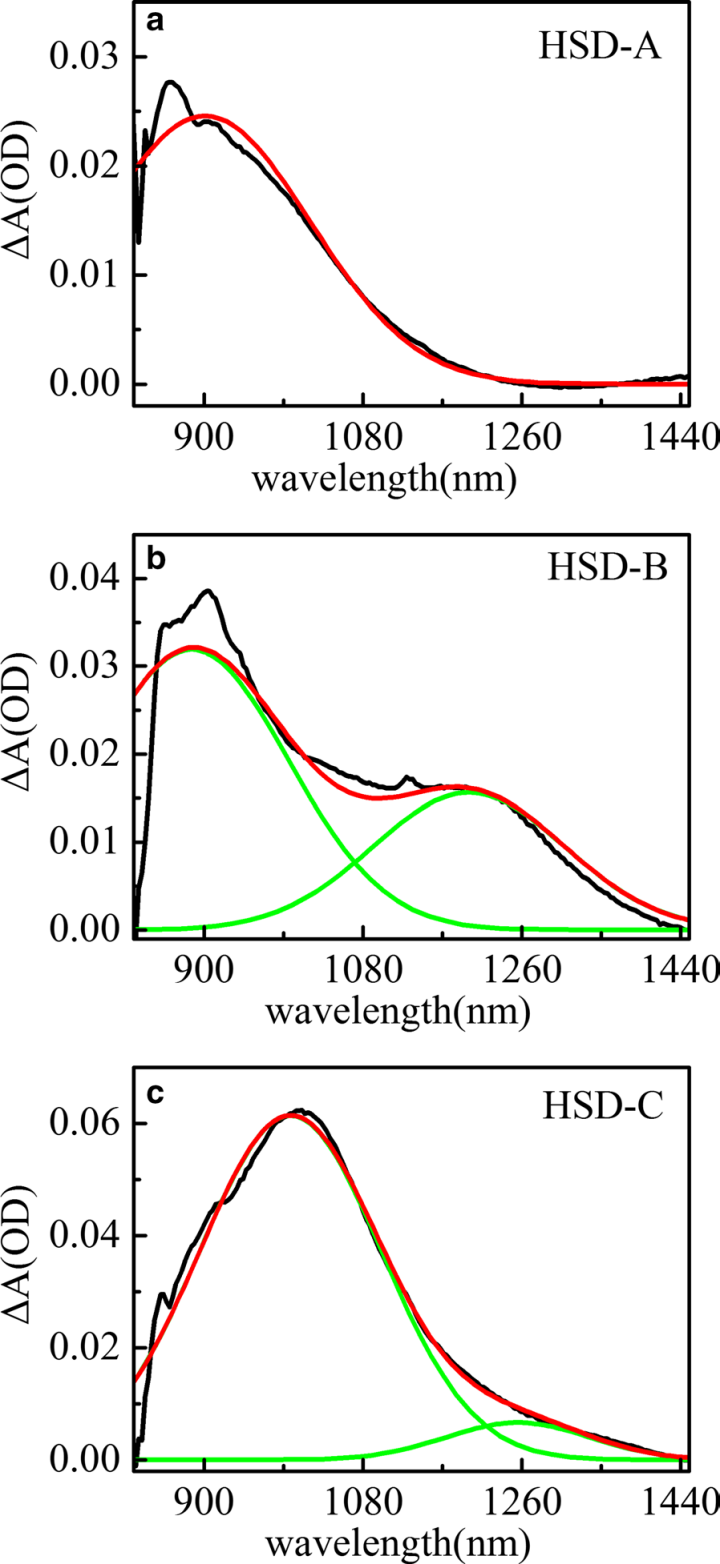
Transient absorption spectral peak-fitted of HSD-A (**a**) and HSD-B (**b**) and HSD-C (**c**) at the delay time of 2 ps in the infrared range. Black curve represents the absorption spectrum of the samples at 2 ps, red curve is the fitted absorption spectrum, and the green is the spectral signal identified in the spectrum

Figure 4 exhibits the temporal evolution of differential absorption spectra of the three HSD polymers on ultrafast timescale. In the 490–780 nm range, excitation at low photon energy induces a broad positive signal in the 700–780 nm spectral region which rises promptly with the negative signal in the 490–600 nm spectral region as GSB. We assign the broad positive signal from 700 to 780 nm to exciton (EX) state absorption is attributed as follows. Firstly, its lifetime is consistent with the lifetime of other excitons in the isolated polymers in the literature of 500–1000 ps [35] and is much shorter than the lifetime of the charge transfer (CT) state and the charge separated (CS) state, which has a time scale greater than a couple of nanoseconds. Secondly, it has a similar dynamic trend to GSB in the first few hundred picoseconds. Another positive signal (peaked at about 600 nm) appears after a few picoseconds, which corresponds to the formation of the mobile carrier (MC) state. Since the about 600 nm spectra could be reasonably chalked up to the fact that the superposition of the GSB and MC absorption in the transient absorption experiments. The initial negative signal at 600 nm is owing to the fact that the signal of the GSB is much stronger than the signal of MC absorption. As the delay time increases, the positive TA feature appears when MC absorption is stronger than that of the GSB. In addition, the reason for the sunken at 650 nm is that the unstable excited state returns to ground state due to the stimulated emission (SE) and the spectral consistence with the steady-state fluorescence. In NIR ranges, it can be seen that the absorption signals of the three samples increased within 1 ps with a peak at about 1 ps and then show a tendency to be attenuated. Interestingly, the shape and attenuation trend of the absorption signals of the three samples are different. In order to analyze these differences in more detail, we performed peak fitting on the infrared spectra, and the results are shown in Fig. 5.

**Fig. 6:**
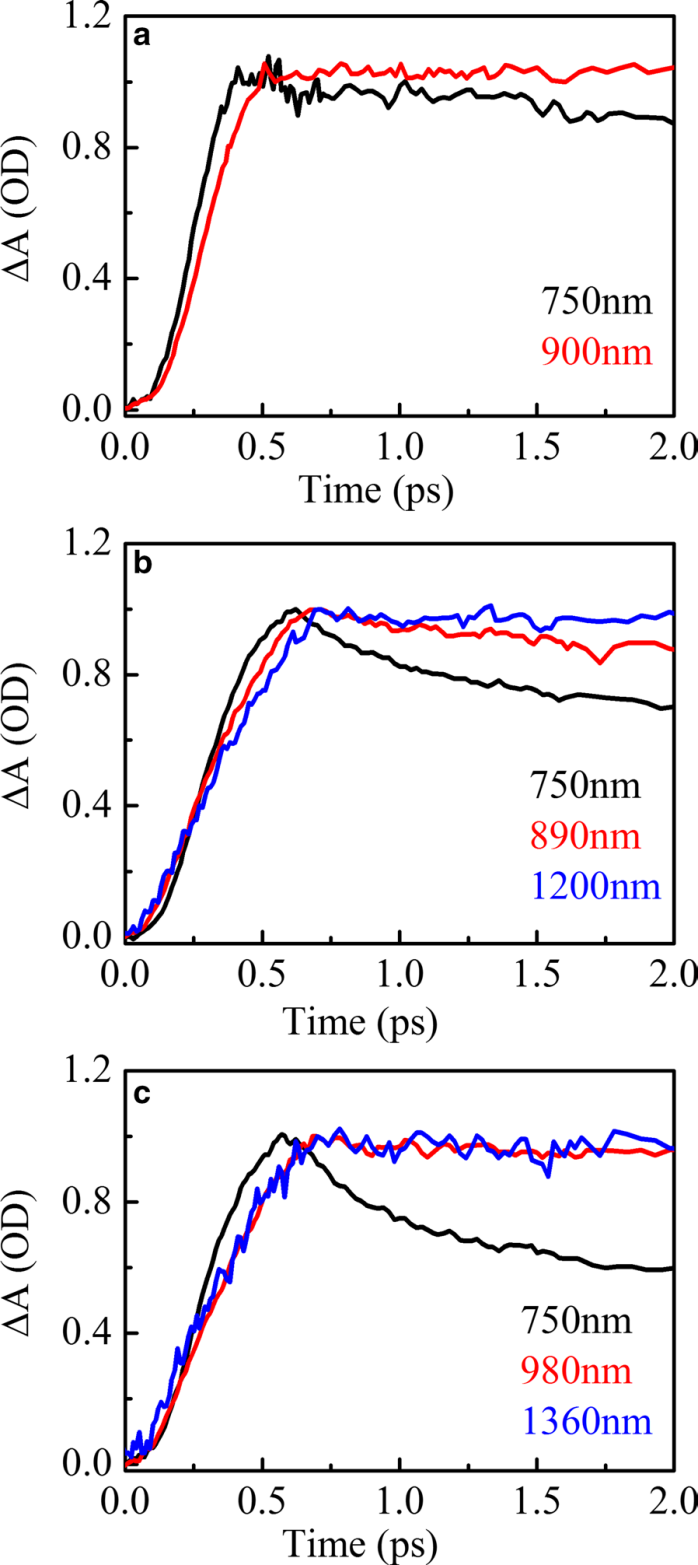
Transient absorption EX (black) and CT (red) and CT′ (blue) kinetics for HSD-A (**a**) and HSD-B (**b**) and HSD-C (**c**), exhibiting the EX decay simultaneously with the increase in CT and CT′

Figure 5 shows the transient absorption spectra peak fitting of HSD-A (a) and HSD-B (b) and HSD-C (c) at the delay time of 2 ps in the infrared range. In HSD-A, the spectra could be well fitted by one component analysis, while in HSD-B and HSD-C, these spectra could be greatest approximated by two different component analyses. This means that HSD-B and HSD-C have one more absorption signal in the infrared range than HSD-A dose, which is due to the addition of the thiophene bridge. The addition of the thiophene bridge expands the absorption range of the polymers in NIR, enabling HSD-B and HSD-C to have a new absorption peak near 1200 nm. In the early time, these two positive signals have a rise time closely related to the attenuation of the EX peaks as shown in Fig. 6, which shows that these positive signals are directly generated by the EX states. The positive signals near 900 nm in all three samples could be allocated to the intramolecular charge transfer (CT) state. In this state, the excitons split into hole-electron pairs, and the hole-electron pairs are still close enough to generate Coulomb gravity [36, 37]. Another new positive signals near 1200 nm only present in HSD-B and HSD-C and also are accompanied by the attenuation of EX in the early period, but have a different attenuation trend from the CT state under a long time window. This is a new exciton dissociation channel, and we regard it as the CT′ state. Because it has similar characteristics to the CT state, but the decay dynamics is different from the CT state.

**Fig. 7:**
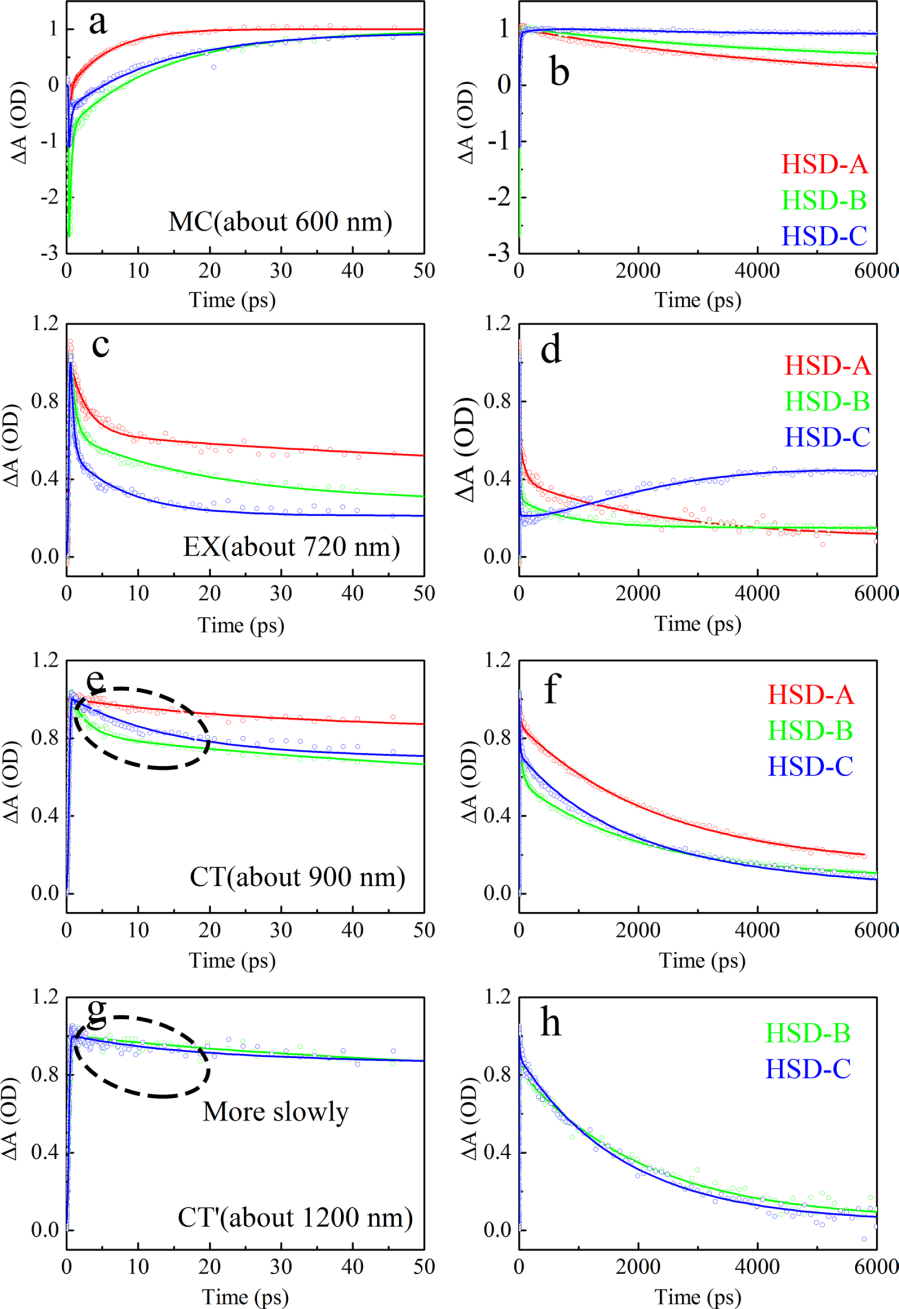
Kinetics fit of all transient spectral characteristics of three samples. The figure shows the kinetics of 50 ps (**a**, **c**, **e**) and 5000 ps (**b**, **d**, **f**). Fits are for MC (**a**, **b**), EX (**c**, **d**), CT (**e**, **f**), CT′ (**g**, **h**) spectrum characteristics

As shown in Fig. 7, the dynamic curves of MC state, EX state, CT state, and CT′ in HSD-A, HSD-B, and HSD-C are extracted and fitted, respectively, which represent the time evolution of different components. The fitting formula for the dynamic curves is ∆A(t) = a_1_exp(− t/τ_1_) + a_2_exp(− t/τ_2_) + ··· + a_n_exp(− t/τ_n_), where a_1_, a_2_, …a_n_ is amplitudes, τ_1_, τ_2_, …, τ_n_ correspond to time constants [38, 39]. Table 1 lists the fitted time components and relative amplitudes. For comparison, the maximum amplitudes are normalized. It can be clearly seen from the data of MC state fitting (Fig. 7a, b) that the carrier generation speed of HSD-A is the fastest one. Its formation life is 6.43 ps, which is shorter than 12.6 ps of HSD-B and 8.41 ps of HSD-C. However, the carriers of HSD-A decay rapidly after being formed, while HSD-B and HSD-C have an additional slow rise process with the time constant of 28.8 ps and 26.4 ps, respectively. This could make the carriers in HSD-A more difficult to be captured, which is likely to be one of the reasons for the lower PCE of the devices. In the EX state (Fig. 7c, d), the decay trends of the three samples are obviously different. HSD-A has a significantly longer decay life, so the exciton splitting is relatively slow. In HSD-B and HSD-C, there are three decay lifetimes in 1 ns, one femtosecond (0.712 ps for HSD-B, 0.408 ps for HSD-C), and one picosecond (18.4 ps for HSD-B, 7.96 ps for HSD-C) lifetimes represent the transition of EX state to other states. The longer lifetime of hundreds of picoseconds (735 ps for HSD-B, 627 ps for HSD-C) is of the same order of magnitude as previously reported exciton lifetime of isolated P3HT. Therefore, it can be considered that $\uptau _{3}^{{{\text{EX}}}}$ in the EX fitting is most likely to be the exciton lifetime without the transition process [35, 40]. However, in a long time window, the exciton recombination occurs in HSD-C. The CT kinetics (Fig. 7e, f) of these three samples are best fitted by three lifetime components. The short increasing time constant, $\uptau _{1}^{{{\text{CT}}}} < 1$ ps, is closely related to the concurrent decay lifetime of the EX state, which means the transition from EX state to CT state. Similarly, there is also a good relevance between the decay lifetime of CT state $\uptau _{2}^{{{\text{CT}}}}$ and the increasing lifetime of the carrier state $\uptau _{2}^{{{\text{MC}}}}$, indicating the transition from CT state to MC state. Comparing the CT state decay lifetimes of the three samples, it can be found that the CT state decay lifetimes of HSD-B is significantly shorter than those of HSD-A and HSD-C, indicating that HSD-B has a faster CT state decay rate. The CT′ state (Fig. 7g, h) of HSD-B and HSD-C exhibits different dynamic curves from the CT state in the 50 ps interval. It has a longer transition lifetime $\uptau _{2}^{{{\text{CT}}^{{\prime }} }}$, which is in good correlation with $\uptau _{3}^{{{\text{MC}}}}$, which represents the transition from CT′ state to MC state.

**Table 1:** Transient absorption kinetics fitting parameters [lifetime (τ) and weight coefficient (A)] of MC, EX, CT, and CT′ state

Sample	State	τ_1_/ps (A)	τ_2_/ps (A)	τ_3_/ps (A)	τ_4_/ps (A)	τ_5_/ps (A)
HSD-A	MC	0.183 (− 0.46)	6.43 (− 0.28)	–	4950 (0.27)	
HSD-B	MC	0.304 (− 0.51)	12.6 (− 0.29)	28.8 (− 0.03)	≥ 6000 (0.18)	
HSD-C	MC	0.296 (− 0.39)	8.41 (− 0.24)	26.4 (− 0.16)	≥ 6000 (0.27)	
HSD-A	EX	2.41 (0.38)	84.4 (0.24)	2450 (0.29)	≥ 6000 (0.09)	
HSD-B	EX	0.712 (0.49)	18.4 (0.27)	735 (0.12)	≥ 6000 (0.12)	
HSD-C	EX	0.408 (0.19)	7.96 (0.06)	627 (0.03)	4410 (− 0.35)	≥ 6000 (0.37)
HSD-A	CT	0.132 (− 0.54)	8.61 (0.07)	3250 (0.39)		
HSD-B	CT	0.155 (− 0.53)	15.8 (0.19)	2790 (0.29)		
HSD-C	CT	0.215 (− 0.51)	12.3 (0.17)	2240 (0.32)		
HSD-B	CT′	0.289 (− 0.51)	30 (0.1)	2440 (0.39)		
HSD-C	CT′	0.176 (− 0.52)	19.6 (0.08)	2050 (0.4)		

**Fig. 8:**
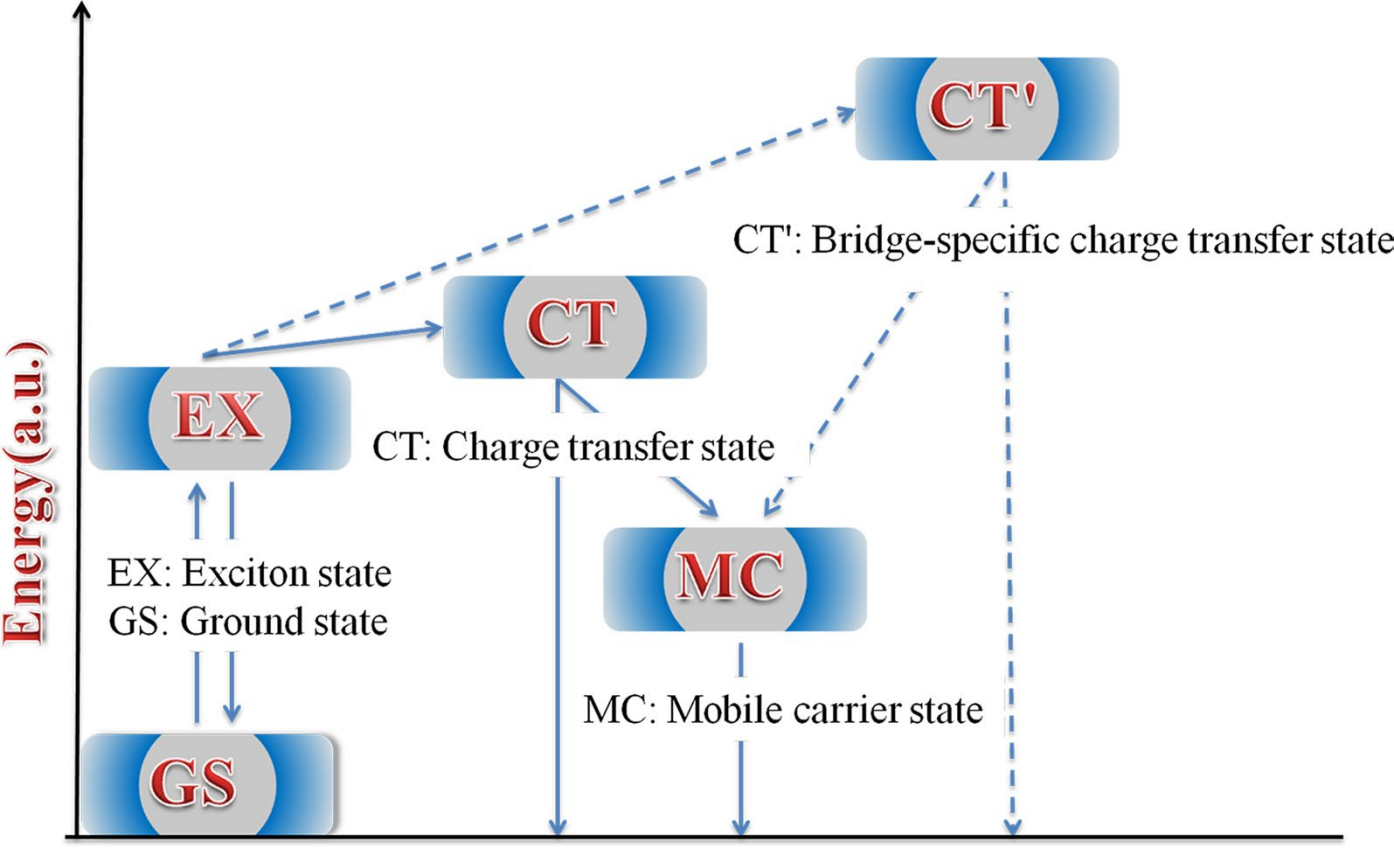
Schematic illustration of the states and pathways in exciton splitting dynamics of the HSD polymers in solution

A schematic of the simplified energy diagram for exciton relaxation pathways is proposed in Fig. 8. The difference in local conformation in the polymers leads to energy variation in different states. Therefore, these states show different TA characteristics. In HSD polymers, MC state, EX state, CT state, and CT′ state are inevitably proposed to give a more reasonable explanation to the exciton relaxation mechanism. Excitons generated after light excitation will quickly split into CT state and CT′ state. In these states, the excitons split into the hole–electron pairs and are still close enough to experience a Coulombic attraction. With the time delay, the electron–hole of CT state and CT′ state will continue to split into a more stable MC states. Importantly, we found that the CT′ state only exists in HSD-B and HSD-C with thiophene bridges, which adds a new exciton splitting channel to the HSD polymers. This will result in higher electron capture rates for HSD-B and HSD-C, which is consistent with the higher PCE of HSD-B and HSD-C as compared to HSD-A. Meanwhile, the fact that the PCE of HSD-C is lower than that of HSD-B could be reasonably explained as follows: (a) the addition of two thiophenes as π bridge might increase the spatial separation between D and A, resulting in nongeminate recombination in HSD-C. (b) As seen from Fig. 5, the proportion of CT′ state of HSD-B is significantly higher than that of HSD-C, which determines the whole PCE of HSD-B. Therefore, CT′ state in HSD polymers is critical for higher electron capture capability to result in higher PCE of HSD polymer devices.

## Conclusions

On balance, we used a combination of steady-state and transient absorption spectroscopy to study the impact of π bridges on HSD copolymers. It was found that the addition of thiophene unit as π bridge will lead to the red shift of steady-state absorption spectrum. Meanwhile, the transient absorption data indicate that HSD-B and HSD-C with thiophene unit as π bridge had an additional CT′ state with the TA fingerprint peak at 1200 nm, which add a new exciton dissociation channel for HSD polymers. The presence of the CT′ state makes the polymer to be advantageous to photoelectric conversion. Among the three HSD polymers studied in this work, only HSD-B and HSD-C containing π bridge have CT′ states. Therefore, we believe that the existence of π bridges promotes the generation of CT′ states. However, the π bridge cannot be too long. For example, the HSD-C polymer with two thiophenes as the π bridge resulting in nongeminate recombination due to the existence of the too long π bridge and affects the ratio of CT′ states. In addition, we also elucidated the relaxation pathways of exciton by analyzing dynamic fit of all transient spectral characteristics. These findings provide important photophysical information for improving power conversion efficiencies of the conjugated polymers and further development of organic solar cells.

## Data Availability

The datasets used and analyzed in the current study can be obtained from the corresponding authors upon reasonable request.

## Abbreviations

D-π-A: Donor-π-acceptor
TA: Transient absorption
CT′: Bridge-specific charge transfer state
OPV: Organic photovoltaic
PCE: Power conversion efficiencies
NIR: Near infrared
BHJ: Bulk heterojunction
TOPAS: Optical parametric amplifier
WLC: White-light continuum
PL: Photoluminescence
VIS: Visible
GSB: Ground state bleaching
ESA: Excited state absorption
EADS: Evolution-associated difference spectra
EX: Exciton state
CT: Charge transfer state
CS: Charge separated state
MC: Mobile carrier state
SE: Stimulated emission
